# Tissue-specific localization of tick-borne pathogens in ticks collected from camels in Kenya: insights into vector competence

**DOI:** 10.3389/fcimb.2024.1382228

**Published:** 2024-04-18

**Authors:** Rua Khogali, Armanda Bastos, Joel L. Bargul, Dennis Getange, James Kabii, Daniel Masiga, Jandouwe Villinger

**Affiliations:** ^1^ International Centre of Insect Physiology and Ecology (icipe), Nairobi, Kenya; ^2^ Department of Zoology and Entomology, University of Pretoria, Pretoria, South Africa; ^3^ Department of Parasitology, Faculty of Veterinary Medicine, University of Khartoum, Khartoum North, Sudan; ^4^ Department of Veterinary Tropical Diseases, University of Pretoria, Pretoria, South Africa; ^5^ Department of Biochemistry, Jomo Kenyatta University of Agriculture and Technology (JKUAT), Nairobi, Kenya; ^6^ School of Life Sciences, University of KwaZulu-Natal, Durban, South Africa

**Keywords:** dromedary camels, tick tissues, Rhipicephalus pulchellus, Amblyomma gemma, Hyalomma dromedarii, Hyalomma rufipes, Ehrlichia, Rickettsia

## Abstract

**Background:**

Tick-borne pathogen (TBP) surveillance studies often use whole-tick homogenates when inferring tick-pathogen associations. However, localized TBP infections within tick tissues (saliva, hemolymph, salivary glands, and midgut) can inform pathogen transmission mechanisms and are key to disentangling pathogen detection from vector competence.

**Methods:**

We screened 278 camel blood samples and 504 tick tissue samples derived from 126 camel ticks sampled in two Kenyan counties (Laikipia and Marsabit) for *Anaplasma, Ehrlichia, Coxiella, Rickettsia, Theileria*, and *Babesia* by PCR-HRM analysis.

**Results:**

*Candidatus* Anaplasma camelii infections were common in camels (91%), but absent in all samples from *Rhipicephalus pulchellus, Amblyomma gemma, Hyalomma dromedarii*, and *Hyalomma rufipes* ticks. We detected *Ehrlichia ruminantium* in all tissues of the four tick species, but *Rickettsia aeschlimannii* was only found in *Hy. rufipes* (all tissues). *Rickettsia africae* was highest in *Am. gemma* (62.5%), mainly in the hemolymph (45%) and less frequently in the midgut (27.5%) and lowest in *Rh. pulchellus* (29.4%), where midgut and hemolymph detection rates were 17.6% and 11.8%, respectively. Similarly, in *Hy. dromedarii, R. africae* was mainly detected in the midgut (41.7%) but was absent in the hemolymph. *Rickettsia africae* was not detected in *Hy. rufipes*. No *Coxiella, Theileria*, or *Babesia* spp. were detected in this study.

**Conclusions:**

The tissue-specific localization of *R. africae*, found mainly in the hemolymph of *Am. gemma*, is congruent with the role of this tick species as its transmission vector. Thus, occurrence of TBPs in the hemolymph could serve as a predictor of vector competence of TBP transmission, especially in comparison to detection rates in the midgut, from which they must cross tissue barriers to effectively replicate and disseminate across tick tissues. Further studies should focus on exploring the distribution of TBPs within tick tissues to enhance knowledge of TBP epidemiology and to distinguish competent vectors from dead-end hosts.

## 1 Introduction

Ticks are obligatory hematophagous ectoparasites of many vertebrate species, including both domestic and wild animals (Villar et al., 2017; Michalik et al., 2020). They are one of the major vectors of emerging and re-emerging diseases, including bacterial diseases such as Q fever, rickettsiosis, ehrlichiosis, and anaplasmosis, protozoal diseases like babesiosis and theileriosis, and viral diseases such as Crimean-Congo hemorrhagic fever (Jongejan and Uilenberg, 2004; de la Fuente et al., 2017). Ticks cause considerable economic losses to livestock farmers in tropical regions due to infected wounds, blood loss through tick infestation and pathogen transmission (Parola and Raoult, 2001; de la Fuente et al., 2008). Kenya has the third largest population of the one-humped, dromedary camels (*Camelus dromedarius)* in Africa (Hughes and Anderson, 2020) with an estimated camel population of more that 3 million, approximately 6% of Africa’s camel population (Kagunyu and Wanjohi, 2014; FAO, 2016). An estimated USD 11 million of camel meat and milk is produced annually in Kenya (Collins et al., 2022). In Kenya, several tick-borne bacteria, such as *Candidatus* Anaplasma camelii, *Cadidatus* Ehrlichia regeneryi, *Coxiella burnetii, Ehrlichia ruminatium*, *Ehrlichia chaffensis*, *Rickettsia aeschlimannii*, *Rickettsia africae*, and *Coxiella* endosymbionts, have been detected in different tick species collected from camels (Koka et al., 2017; Kidambasi et al., 2020; Getange et al., 2021; Younan et al., 2021). An understanding of the ecology of these ticks and the prevalence of associated tick-borne pathogens (TBPs) is crucial for efficient surveillance and better control strategies (Boulanger et al., 2019; Zhao et al., 2021). Ticks are competent vectors for a broad range of vector-borne pathogens (Kahl, 2018). Various factors influence this competence, with tick-pathogen interactions being an important aspect (de la Fuente et al., 2017). To elucidate these interactions, insights into the localization and migration of pathogens within ticks is needed and reliant of pathogen detection in different tick tissues. This, in turn, enhances our understanding of TBP transmission and vector competence (Lejal et al., 2019).

Tick fluids and organs play a crucial role in TBP transmission cycles. Ticks inject saliva, produced by tick salivary glands (SGs), into the vertebrate host to maintain the flow of blood during feeding. Therefore, saliva and SGs play an important role in TBP transmission (Šimo et al., 2017; Nuttall, 2019). The midgut (MG) is the initial organ that encounters blood-borne pathogens and provides a barrier which must be penetrated to achieve hemolymph invasion. Thus, the MG barrier influences pathogen survival and colonization inside the tick (Lejal et al., 2019). Hemolymph is a medium for nutrient and cellular metabolite transport and provides protection against pathogens; it is a circulating fluid that bathes the tick’s inner organs (Sonenshine and Roe, 2013; Liu and Bonnet, 2014). Thus, the pathogens must encounter the immunity supplied by the tick’s hemolymph (Bonnet et al., 2018). In competent vectors, pathogens penetrate and invade tick salivary glands and are subsequently transmitted to new hosts via saliva injection during subsequent blood meals (Šimo et al., 2017). This complexity of development within the tick affects TBP transmission (Ueti et al., 2007). According to previous studies, *Anaplasma marginale* is able to replicate inside the SGs and the MG, which act as both early and late barriers for efficient TBP transmission (Ueti et al., 2007; Lejal et al., 2019). *Anaplasma phagocytophilum* and *Borrelia burgdorferi* rapidly multiply and colonize the midgut of feeding tick larvae and decrease during molting. Subsequently, they migrate to the SGs of the nymphs after stimulation by a new blood meal (Zhang et al., 2011; Radolf et al., 2012; Liu and Bonnet, 2014; Coumou et al., 2016). In contrast, *Borrelia afzelii* increases during molting, but decreases during nymph feeding, where spirochetes found in the midgut are infective to vertebrate hosts (Pospisilova et al., 2019). Some rickettsial species initially replicate in the MG and are immediately transported via the hemolymph to the SGs where they proliferate and are subsequently released from the saliva to the host (Socolovschi et al., 2009; Liu and Bonnet, 2014). Conversely, *Babesia bigemina* and *Theileria parva*, both apicomplexan parasites, replicate within the gut lumen as male and female gametes. Subsequently, the zygotes multiply inside the epithelial cell lining of the gut, forming motile kinetes. *Babesia bigemina* kinetes invade the ovaries and SGs, while *T. parva* kinetes invade the SGs during molting to form the sporozoites. Sporozoites maturation occurs in the SGs with transmission through saliva during host feeding (Uilenberg, 2006; Sonenshine and Roe, 2013). Tick-borne encephalitis (TBE) and Crimean-Congo hemorrhagic fever (CCHF) viruses multiply within the MG, then spread to the hemolymph, eventually infecting different tick tissues (Liu et al., 2022). They reach the highest titers in the SGs and reproductive organs (Dickson and Turell, 1992; de la Fuente et al., 2017). Better screening of TBPs at the tick organ/tissue level can lead to improved understanding of specific transmission dynamics (Tack et al., 2012; Pollet et al., 2020).

This study aimed to identify key TBPs in the blood of dromedary camels (*Camelus dromedarius*) of pastoralist communities in Kenya. Camel-associated tick species were assessed at a finer tissue/organ scale (saliva, hemolymph, SGs, and MG) to identify potential mechanisms of pathogen transmission and to disentangle infection status from vector competence. Although the pathogens might be found in both the animal and attached ticks, the presence of TBPs in the tick body, does not indicate that ticks are the efficient vectors. Knowledge of the distribution of TBPs within tick tissues can inform tick-borne disease epidemiology, and in particular, the potential of specific species to be competent vectors or dead-end hosts. To achieve this, we used PCR-based assays to detect TBPs in camel blood and key tick tissues.

## 2 Materials and methods

### 2.1 Study site

**Figure 1:**
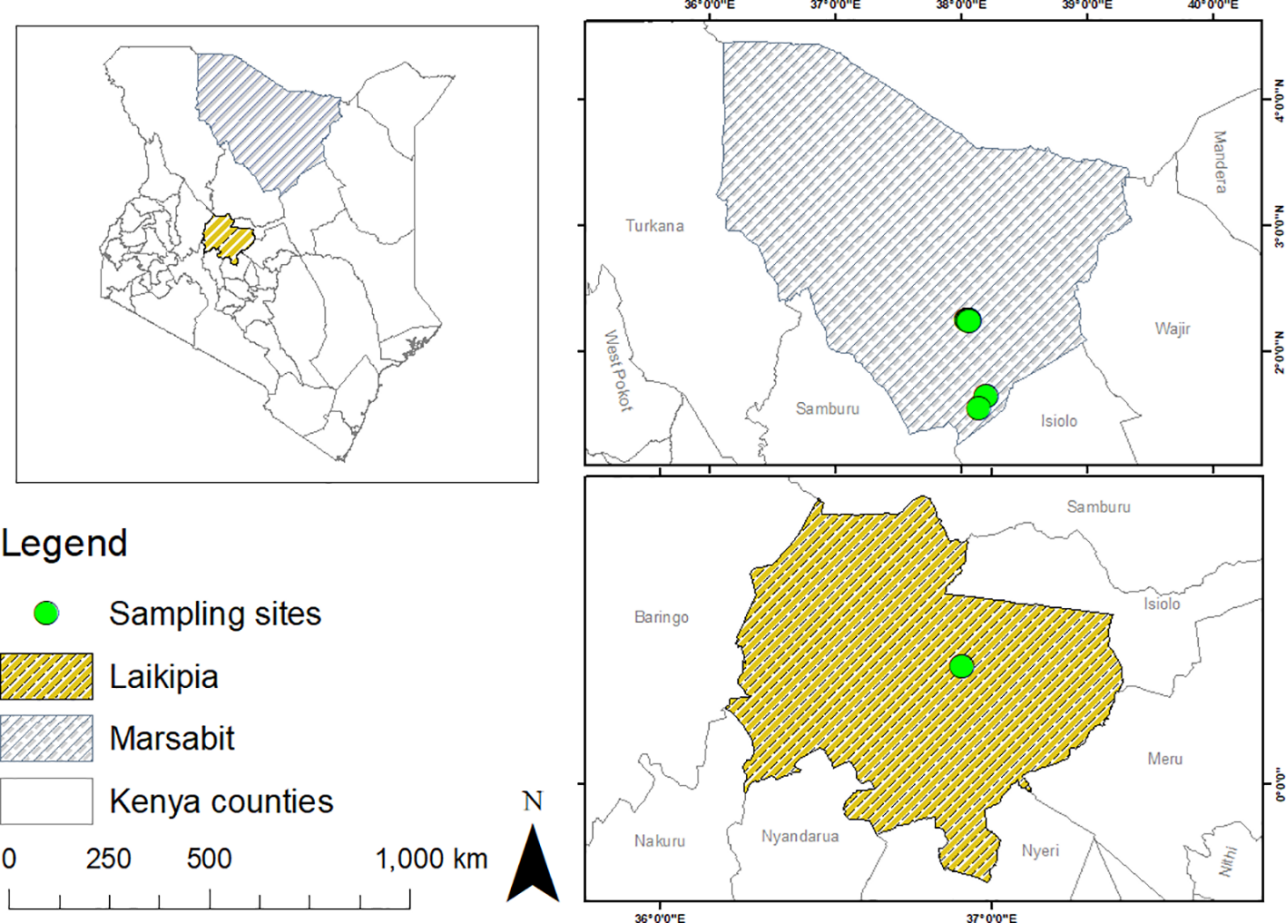
Maps showing the sampling sites where ticks and blood were collected from camels in Laikipia and Marsabit counties. Maps were generated using the open-source software, QGIS v.3.28.4.

This study was conducted in May 2022 in Laikipia County, and in November 2022 in Marsabit County. Laikipia is in the Rift Valley region of Kenya, and occupies an area of about 10,000 km² in size between latitude 0°53’N, 0°16’S and longitude 36°11’E, 37°23’E. The County is classified as a semi-arid region, which is prone to seasonal flooding (Kamau et al., 2021). Livestock species such as camels are kept for milk, meat production, and transportation (Deem et al., 2015; Browne et al., 2017). Marsabit County covers an area of ~66,923 km^2^ located between longitudes 37°57’ and 39°21’E and latitudes 02°45’ and 04°27’ N, borders Wajir and Isiolo counties to the East, and is home to pastoralist camel keepers who predominantly rely on mobile livestock production for their livelihoods (Getange et al., 2021). Samples were obtained from dromedary camels (*Camelus dromedarius*) in camel ranches adjacent to Mpala Research Centre, Laikipia County, and in Laisamis and Badassa in Marsabit County (**Figure 1**).

### 2.2 Ethical approval

The study received ethical approval from the Pwani University Ethics Review Committee (Ref: ERC/EXT/002/2020E) and also obtained a license to conduct the research from Kenya’s National Commission for Science Technology and Innovation (NACOSTI) (Ref: NACOSTI/P/22/16467). Sampling of camels for blood was undertaken by a veterinarian, with care being taken to ensure minimum distress. Prior to livestock sampling, oral consent was obtained from the livestock keepers, as most were unable to read or write. Field assistants from the community assisted in restraining the camels and helped in translating the language from English to the local language spoken by the community members to ensure that they understood the purpose of the study and how it would benefit them.

### 2.3 Blood and tick collection

We collected blood and tick samples from camels in Laikipia and Marsabit counties. We pre-screened the camel blood samples collected at Mpala Research Center in Laikipia, to detect *Anaplasma* spp., prior to sampling ticks from both infected and non-infected camels. Thus, the camel blood was drawn from the jugular veins into 10-mL EDTA vacutainer tubes (BD Vacutainer^®^) using 18-gauge rubber-capped needles. Each tube was labelled with a unique animal identifier, and other parameters for baseline data were recorded in sampling record form, including sex, age, any recent health history, and whether there were any obvious ectoparasites on them (biting flies, keds, ticks). Blood samples in the EDTA vacutainers were transferred into labelled-cryovials and preserved in liquid nitrogen for transportation to *icipe’s* Martin Lüscher Emerging Infectious Diseases (ML-EID) Laboratory in Nairobi, for further analysis. Live ticks were placed in the falcon tubes containing cotton wool wads as stoppers, wrapped with moisturized gauze and placed inside a styrofoam box containing wet towels to maintain optimum relative humidity and ambient temperature during transportation to the ML-EID Laboratory for analysis.

### 2.4 Morphological and molecular identification of ticks

Ticks were morphologically identified to the species level using standard taxonomic keys (Walker et al., 2003) by light microscopy using a Stemi 2000-C microscope (Zeiss, Oberkochen, Germany). In addition, representative individual leg samples of morphologically identified ticks were used for DNA isolation followed by PCR-based molecular identification of tick samples through partial sequencing of the cytochrome oxidase subunit I (COI), 16S ribosomal (r)RNA, and 12S rRNA genes. We performed PCR in 10-µL reaction volumes consisting of 2 µL 5× HOT FIREPol® Blend Master Mix (Solis BioDyne, Tartu, Estonia), 0.5 µL of 10 µM forward and reverse primers (**Table 1**) and ~25 ng DNA template in a ProFlex PCR systems thermocycler (Applied Biosystems, Foster City, CA, USA). The amplification conditions consisted of an initial denaturation at 95°C for 15 min, 35 cycles of denaturation at 95°C for 20 s, annealing at 55°C (for both CO1 and 16S rRNA) and 50°C (for 12 rRNA) for 30s, and extension at 72°C for 30 sec, and a final extension of at 72°C for 7 min.

**Table 1:** Primers used to amplify tick and tick-borne pathogen DNA.

Primer name	5’ to 3’ sequence	Target gene	Amplicon size (bp)	Primer reference
**Tick COI F** **Tick COI R**	ATTCAACCAATCATAAAGATATTGG TAAACTTCTGGATGTCCAAAAAATCA	Tick COI	658 bp	Hebert et al. (2004)
**SR-J14199F** **SR-N14594R**	TACTATGTTACGACTTAT AAACTAGGATTAGATACCC	Tick 12S rRNA	430 bp	Simon et al. (1994)
**Tick16S** **Tick 16S**	AATTGCTGTAGTATTTTGAC TCTGAACTCAGATCAAGTAG	Tick 16S rRNA	450 bp	Brahma et al. (2014)
**Rick-F** **Rick-R**	GAACGCTATCGGTATGCTTAACACA CATCACTCACTCGGTATTGCTGGA	*Rickettsia* 16S rRNA	364 bp	Nijhof et al. (2007)
**120–2788** **120–3599**	AAACAATAATCAAGGTACTGT TACTTCCGGTTACAGCAAAGT	*Rickettsia* ompB	856 bp	Roux and Raoult (2000)
**Trans1** **Trans2**	TGGTATTCTTGCCGATGAC GATCGTAACTGCTTAATAAACCG	*Coxiella* IS1111	687 bp	Hoover et al. (1992)
**Ehlichia16SF** **Ehrlichia16SR**	CGTAAAGGGCACGTAGGTGGACTA CACCTCAGTGTCAGTATCGAACCA	*Ehrlichia* 16S rRNA	200 bp	Tokarz et al. (2009)
**PER1** **PER2**	TTTATCGCTATTAGATGAGCCTATG CTCTACACTAGGAATTCCGCTAT	*Ehrlichia* 16S rRNA	451 bp	Goodman et al. (1996)
**EHR16SD** **1492R**	GGTACCYACAGAAGAAGTCC GGTTACCTTGTTACGACTT	*Anaplasma*/*Ehrlichia*16S rRNA	1030 bp	Parola et al. (2000) & Reysenbach et al. (1992)
**AnaplasmaJF** **AnaplasmaJR**	CGGTGGAGCATGTGGTTTAATTC CGRCGTTGCAACCTATTGTAGTC	*Anaplasma*16S rRNA	300 bp	Mwamuye et al. (2017)
**RLB F** **RLB R**	GAGGTAGTGACAAGAAATAACAATA TCTTCGATCCCCTAACTTTC	*Theileria*/*Babesia*18S rRNA	460-520 bp	Gubbels et al. (1999)
**icd-439F** **icd-514R**	CGTTATTTTACGGGTGTGCCA CAGAATTTTCGCGGAAAATCA	*Coxiella burnetii* isocitrate dehydrogenase gene	76 bp	Klee et al. (2006)

### 2.5 Isolation of tick tissues

We collected four tissues, saliva (SL), hemolymph (HL), salivary glands (SGs), and midgut (MG), from each of 126 ticks using the Stemi 2000-C microscope (Zeiss, Oberkochen, Germany). Firstly, tick mouthparts and the whole bodies were sterilized using 70% ethanol. Using a 10-µL pipette, we collected SL from the mouthparts after injecting the ticks with 2% Pilocarpine HCL behind coxa four, and HL droplets after cutting the legs (Tirloni et al., 2021; Patton et al., 2012). Subsequently, the SGs and MG were isolated as per the published protocol by Edwards et al. (2009). Prior to dissection, the ticks were dipped three times in 1% bleach solution followed by a final rinse in nuclease-free water (Binetruy et al., 2019), SGs and MGs were rinsed with the five sequential droplets of the phosphate-buffered saline to avoid the contamination by the HL. The four samples from each tick were placed separately in well-labeled cryovials containing information on the tissue type, tick species, tick sex, from which they were obtained, and the tick sampling site on the camel host, camel host ID and then stored at -80°C.

### 2.6 DNA extraction

Isolated tick organs (SGs and MG) were homogenized in sterile 1.5-mL Eppendorf tubes containing 750 mg of 2.0-mm stabilized zirconium oxide beads (Biospec, USA) using a Mini-Beadbeater-16 (BioSpec, Bartlesville, OK) to mechanically disrupt the samples for 1 min. Each of the resulting SG and MG homogenates and SL and HL samples were used to extract total DNA/RNA using HighPrep™ Viral DNA/RNA kits (Magbio, Gaithersburg, USA). DNA from camel blood samples was extracted using ISOLATE II genomic DNA kits (Bioline, London, UK) according to the manufacturer’s instructions. In addition, we extracted genomic DNA from the tick legs using the Hot Sodium Hydroxide and Tris (HotSHOT) protocol as described by Truett et al. (2000). We added 40 µL alKaine reagent (25 mM NaOH, 0.2 mM Na_2_EDTA) to 0.2-cm segments of tick legs in sterile 0.5-µL microcentrifuge tubes, heated them at 95°C for 30 min, cooled them at 4°C for 15 min, then added 40 µL of neutralizing buffer (40 mM, Tris pH 5.0).

### 2.7 PCR and high-resolution melting analysis

We screened for TBPs by high-resolution melting (HRM) analysis in Mic-4 PCR Cyclers (Bio Molecular Systems, Upper Coomera, Queensland, Australia) and sequencing thermocyclers in the ML-EID and Molecular Biology and Bioinformatic Unit (MBBU) laboratories at *icipe*, Nairobi, Kenya. For initial screening of TBPs, we used previously published genus-specific primers (Mwamuye et al., 2017) that amplify 16S rRNA gene fragments of *Anaplasma, Ehrlichia*, and *Rickettsia* spp. and transposal elements of *Coxiella burnetii*, and the 18S rRNA gene (reverse line blot primers) of *Babesia* and *Theileria* (**Table 1**). The target genes were amplified in 10-µL PCRs that consisted of 2 µL HOT FIREPol® EvaGreen® HRM mix (Solis BioDyne, Tartu, Estonia), 0.5 µL of 10 µM forward and reverse primers (**Table 1**) and 1 µL of template DNA. We included the existing standard samples as positive controls for *Anaplasma, Ehrlichia, Coxiella, Rickettsia*, *Babesia* and *Theileria* spp. Additionally, a no-template, negative control was included in each PCR run by adding 1 µL nuclease-free water instead of DNA. The PCR assays for detecting *Anaplasma, Ehrlichia, Rickettsia*, *Babesia* and *Theileria* spp. were conducted as previously described by Mwamuye et al. (2017).

To generate longer gene sequences for sequencing and phylogenetic analysis, we re-amplified representatives of samples identified as positive based on their HRM profiles visualized with Mic qPCR machine (Bio Molecular Systems, Upper Coomera, Queensland, Australia). *Anaplasma* spp. and *Ehrlichia* spp., were re-amplified using primer sets EHR16SD-1492R and PER1-PER2, respectively, targeting 16S rRNA genes. *Rickettsia*-positive samples were tested further using rickettsial outer membrane protein B (*ompB*) gene primers. These PCRs were run in a ProFlex 3 Real-Time PCR System (Applied Biosystems, Foster City, CA, USA) in 20-µL final reaction volumes consisting of 4 µL of 5x HOT FIREPol® Blend Master Mix (Solis BioDyne, Tartu, Estonia), 2 µL of templates and 1 µl of 10 µM primers. We used the cycling conditions described by Getange et al. (2021). To purify the positive samples, we used the ExoSAP-IT PCR Product Clean-up kit (Affymetrix, Santa Clara, CA, USA) and submitted these for Sanger sequencing to Macrogen (Amsterdam, The Netherlands).

### 2.8 Phylogenetic analysis and statistical analysis

We edited, trimmed, and MAFFT-aligned all sequences obtained from ticks and pathogens using Geneious Prime software v. 2020.2.2 (created by Biomatters, Auckland, New Zealand) (Kearse et al., 2012). Nucleotide sequences were queried against known annotated sequences in the GenBank nr database of NCBI (http://www.ncbi.nlm.nih.gov) using BLAST nucleotide searches (Altschul et al., 1990). Maximum-likelihood phylogenies were inferred for each gene in PhyML v. 3.0 using the Akaike information criterion for automatic model selection (Guindon et al., 2010). Phylogenetic trees were visualized in FigTree 1.4.4 (Rambaut, 2020). Statistical analyses were carried out using R statistical software version 4.1.2 (R Core Team, 2021). We utilized Spearman’s correlation analysis to determine correlations between infections in different tick tissues (saliva, hemolymph, salivary glands, and midgut). The prevalence of the pathogens is presented as a percentage and corresponds to detection rates in infected ticks, tick tissues and camels.

## 3 Results

### 3.1 Collection and identification of ticks

**Figure 2:**
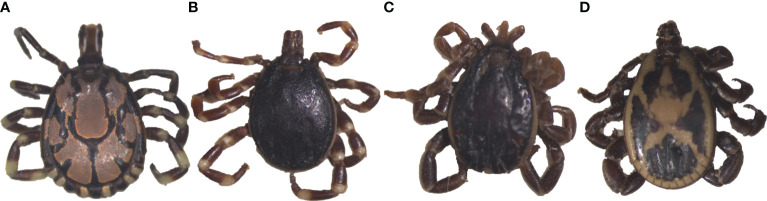
Images of representative adult male ticks collected from camels in Laikipia and Marsabit counites. **(A)** *Amblyomma gemma*, **(B)** *Hyalomma rufipes*, **(C)** *Hyalomma dromedarii*, and **(D)** *Rhipicephalus pulchellus*. The images were captured using a Stemi 2000-C microscope (Zeiss, Oberkochen, Germany), a digital microscope, connected to an Axio-cam ERc 5s camera (Zeiss).

**Figure 3:**
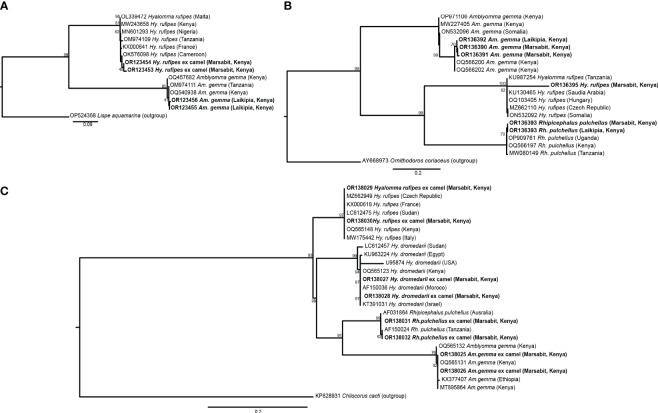
Maximum likelihood phylogenies of representative gene sequences amplified from ticks collected from camels in Laikipia and Marsabit counties. **(A)** tick CO1, **(B)** tick16S rRNA, and **(C)** tick 12S rRNA gene sequences. The study sequences, along with their respective GenBank accessions, are highlighted in bold. The bootstrap values at the nodes are the indicating percentage agreement from 1000 replicates. The branch length scale represents the substitution per site. Trees are rooted to outgroup sequences indicated in brackets.

We collected a total of 278 camel blood samples (53 in Laikipia, 225 in Marasbit) and 1778 adult ticks from three camel herds in Laikipia and ten camel herds in Marsabit. Ticks were morphologically identified as *Amblyomma gemma*, *Rhipicephalus pulchellus, Hyalomma dromedarii* and *Hyalomma rufipes* (**Table 2**). Photos of representative adult male tick species collected during the study illustrate their morphological characteristics (**Figure 2**). The sequences (12S rRNA, 16S rRNA, and CO1) of all four tick species sampled share 98-100% sequence identity with reference sequences from the nr GenBank database, except for the *Hy. rufipes* 16S rRNA gene sequences, which shared only 94-95% with reference sequences, despite sharing >99% identity based on their COI and 12S rRNA gene sequences. All study sequences have been deposited into the GenBank database (12S rRNA gene accessions OR138025-OR138032; 16S rRNA gene accessions OR136390-OR136395; CO1 gene accessions OR123453-OR123456). The maximum likelihood phylogenetic relationships are shown in **Figure 3**. Only the 12S rRNA primers amplified sequences from all tick species.

**Table 2:** Number of tick species collected from camels in Laikipia and Marsabit counties.

Tick species	Number of ticks			County
	Total	Males	Females	
*Amblyomma gemma*	227 (26.8%)	130	97	Badassa, Marsabit
*Rhipicephalus pulchellus*	290 (34.2%)	160	130	
*Hyalomma dromedarii*	187 (22.1%)	116	71	
*Hyalomma rufipes*	143 (16.9%)	88	55	
**Total**	**847**			
*Hyalomma dromedarii*	214 (34.8%)	106	108	Laisamis, Marsabit
*Hyalomma rufipes*	401 (65.2%)	207	194	
**Total**	**615**			
*Amblyomma gemma*	14 (4.1%)	8	5	Mpala, Laikipia
*Rhipicephalus pulchellus*	301 (95.9%)	147	154	
*Hyalomma rufipes*	1(0.3%)	0	1	
**Total**	**316**			

Bold fonts indicate the total numbers of ticks per county.

### 3.2 Pathogen detection in camel blood

**Figure 4:**
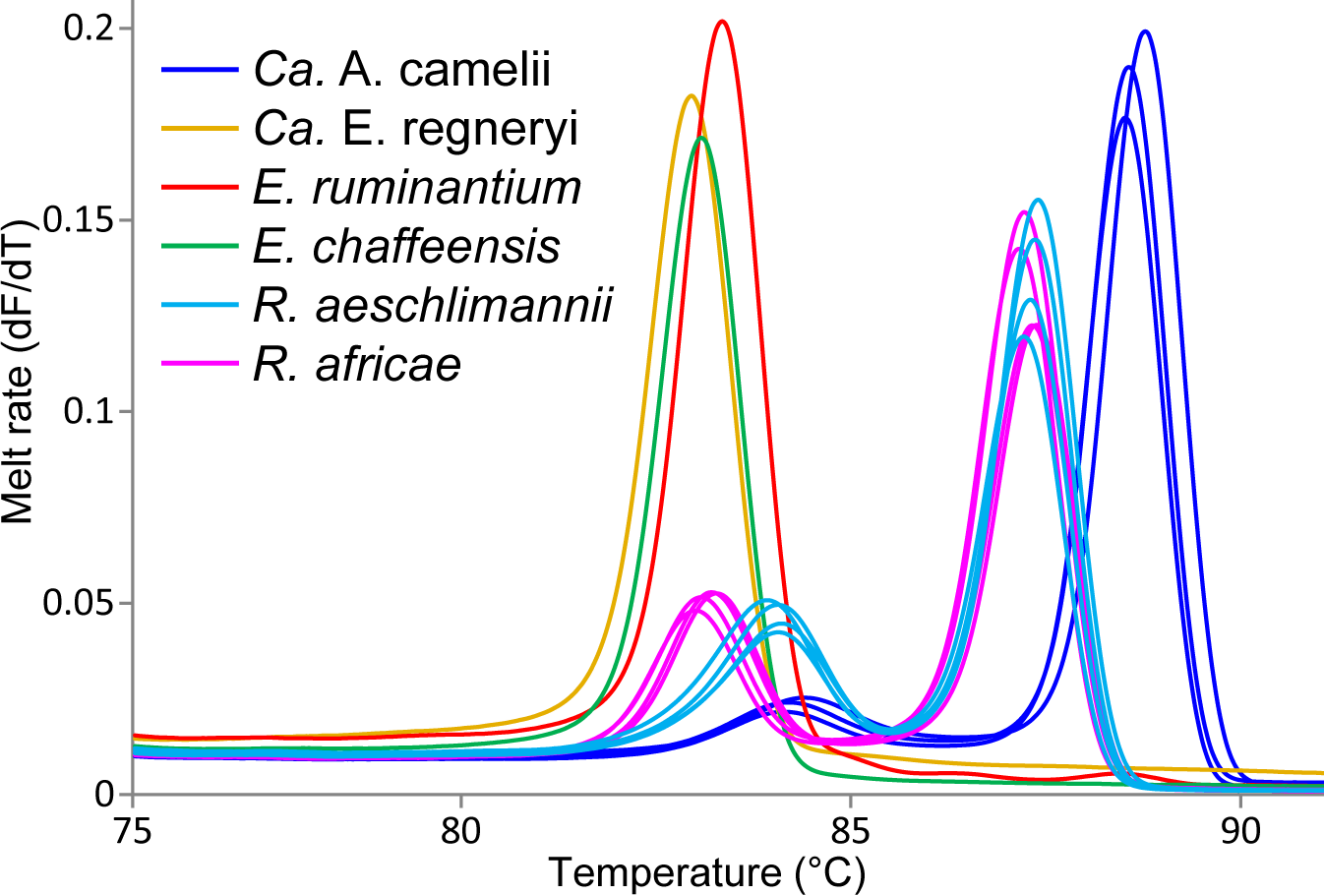
Melt rate profiles of representative tick-borne pathogen PCR products amplified from camel blood and tick tissues. Melt rates are represented as a change in fluorescence with increasing temperature (dF/dT).

**Figure 5:**
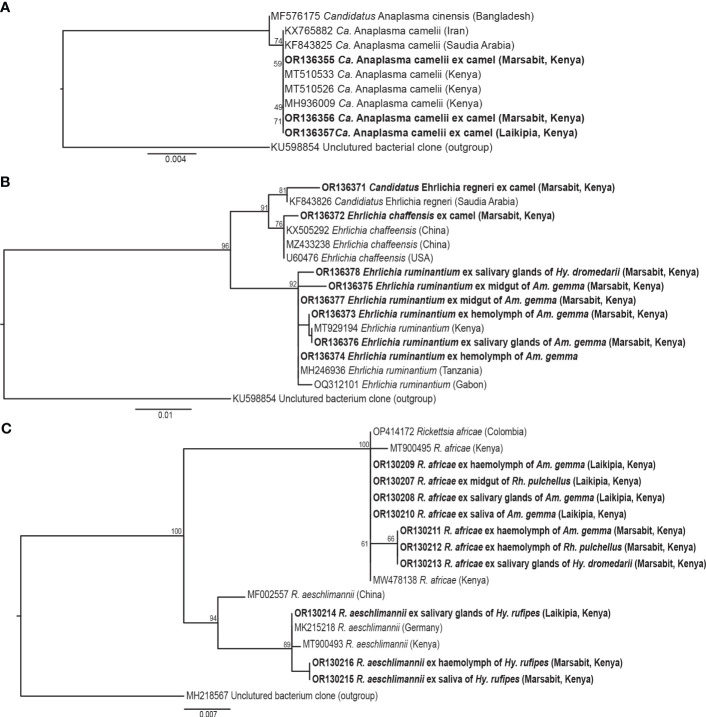
Maximum likelihood phylogenies of representative gene sequences amplified from tick-borne pathogens in tick tissues of camel ticks collected in Laikipia and Marasbit counties. **(A)** Anaplasamataceae 16S rRNA, **(B)** *Ehrlichia* 16S rRNA, and **(C)** *Rickettsia ompB* gene sequences. The study sequences, along with their respective GenBank accessions, are highlighted in bold. The bootstrap values at the nodes are the indicating percentage agreement from 1000 replicates. The branch length scale represents the substitution per site. Trees are rooted to outgroup sequences (indicated in brackets).

We identified *Ca.* Anaplasma camelii, *E. chaffeensis*, and *Ca.* Ehrlichia regneryi in camel blood samples. Unique HRM profiles of representative samples with these infections are shown in **Figure 4**. No *Coxiella burnetii*, *Theileria* or *Babesia* were detected in the blood samples. We found higher *Ca.* Anaplasma camelii infection rates in Laisamis (76.5%) and Badassa (59.6%) within Marsabit County than in Mpala, Laikipia (39.6%). In Badassa one camel was infected with *E. chaffeensis* and one was infected with *Ca.* Ehrlichia regneryi (**Table 3**). The 1030-bp *Ca.* A. camelii 16S rRNA gene sequences obtained in this study (GenBank accessions: OR136355-OR136357) shared over 99% identity with reference *Ca.* A. camelii sequences from Kenya (GenBank accessions: MT510533, MT510526, MH93009), Iran (GenBank accession KX765882), and Saudia Arabia (GenBank accession KF843825). The 451-bp *Ca.* Ehrlichia regneryi 16S rRNA gene sequence (GenBank accession OR136371) obtained also shared over 99% identity with a reference *Ca.* Ehrlichia regneryi sequence from Saudia Arabia (GenBank accessions KF843826), while the 451-bp *E. chaffeensis* sequence (GenBank accession OR136372) obtained shared 100% identity with reference *E. chaffeensis* sequences from China (GenBank accessions KX505292, MZ433238), and the USA (GenBank accession U60476). The maximum likelihood phylogenetic relationships are shown in **Figure 5**.

**Table 3:** Prevalence of tick-borne pathogens detected in camel blood in Laikipia and Marsabit counties.

Bacterial pathogen	Total number of camels tested	Prevalence in camels (%)	Sampling location
***Ca.* Anaplasma camelii**	53	21 (39.6%)	Mpala, Laikipia
	119	91 (76.5%)	Laisamis, Marsabit
	99	59 (59.6%)	Badassa, Marsabit
***Ca.* Ehrlichia regneryi**	99	1 (1.0.1%)	Badassa, Marsabit
***Ehrlichia chaffeensis***	99	1 (1.0.1%)	Badassa, Marsabit

### 3.3 Pathogens detected in ticks

Out 1778 ticks collected, we screened for TBPs in the tissues only 126 adult ticks, 108 from Marsabit and 18 from Laikipia (**Table 4**). The number of ticks that we could fully dissect was limited by time constrains, especially for saliva collection, while the ticks were still alive. Consequently, a total of 504 tick tissues were screened. We detected the DNA of *Rickettsia africae*, *Rickettsia aeschlimannii*, and *Ehrlichia ruminantium*. No *Coxiella burnetti*, *Theileria*, or *Babesia* were detected in any of the ticks collected. Similarly, no *Ca*. A. camelii was detected in the ticks, despite having fed on infected camels. Representative positive samples with unique 16S rRNA HRM melt profiles of *R. africae*, *R. aeschlimannii* and *E. ruminantium* are shown in **Figure 4**. The 856-bp *R. africae* ompB gene sequences (GenBank accessions OR130207- OR130213) obtained shared >99% identity with reference sequences from Kenya (GenBank accessions MT900495 and MW478138) and Colombia (GenBank accession OP414172). The *R. aeschlimannii* sequences (GenBank accessions OR130214, OR130215) shared >98% identity with reference sequences from Kenya (GenBank accession MT900493), Germany (GenBank accession MK215218), and China (GenBank accession MF002557). The 451-bp *E. ruminantium* 16S rRNA gene sequences (GenBank accessions OR136373-OR136378) obtained shared over 99% identity with the reference sequences from Kenya (GenBank accession MT929194), Tanzania (GenBank accession MH246936), and Gabon (GenBank accession OQ312101). The maximum likelihood phylogenetic relationships are shown in **Figure 5**.

**Table 4:** Tick-borne pathogens detected in different tick species collected from camels.

Bacterial pathogen	Tick species	Number of ticks tested	Number of males and females		Number of infected ticks (%)
			Males	Females	
***Rickettsia*** ***africae***	*Am.* *gemma*	40	9	31	**25** (62.5%)
	*Rh.* *pulchellus*	34	12	22	**10** (29.4%)
	*Hy. dromedarii*	36	17	19	**18** (50%)
***Rickettsia aeschlimannii***	*Hy.* *rufipes*	16	1	15	**15** (93.8%)
***Ehrlichia*** ***ruminantium***	*Am.* *gemma*	40	9	31	**14** (35%)
	*Rh.* *pulchellus*	34	12	22	**10** (29.4%)
	*Hy. dromedarii*	36	17	19	**11** (30.5%)
	*Hy.* *rufipes*	16	1	15	**6** (37.5%)

Bold fonts indicate the scientific names of the bacteria and the total numbers of infected ticks.

### 3.4 Associations of pathogens with specific tick tissues

**Figure 6:**
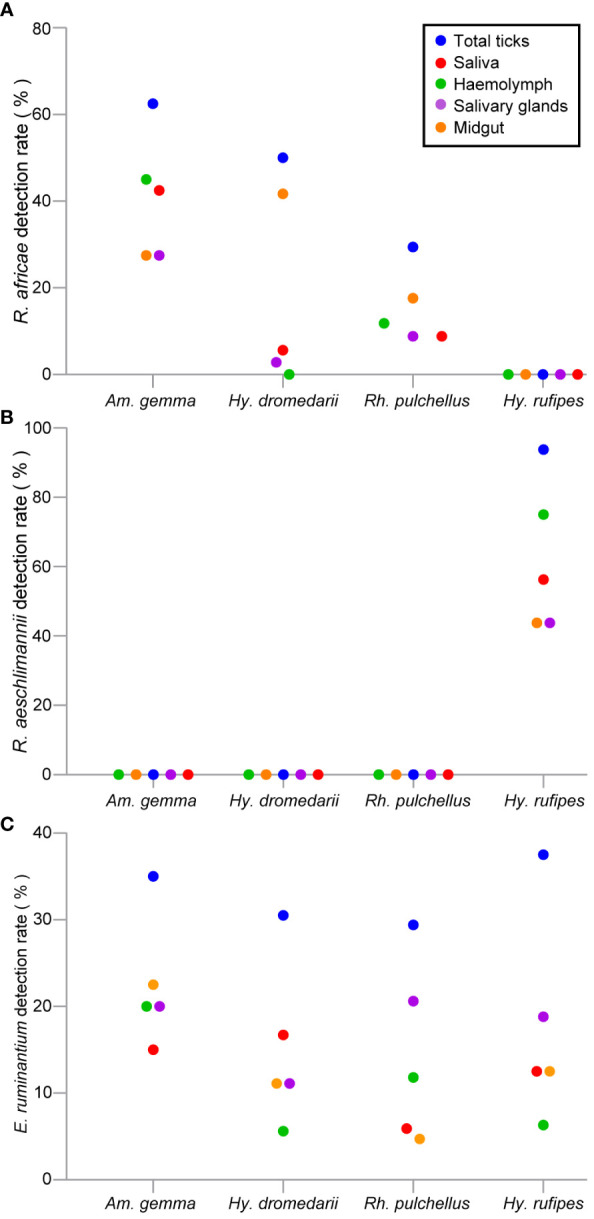
Scattered dot plots showing TBP detection rates (%) in tick tissues across different tick species. **(A)** *R. africae*, **(B)** *R. aeschlimannii*, and **(C)** *E*. *ruminantium.* All tick species were collected from camels in Marsabit and Laikipia counties.

*Rickettsia africae* DNA was most common in *Am. gemma* samples with a detection rate of 62.5%, followed by 50% in *Hy. dromedarii* and 29.4% *Rh. pulchellus* (**Table 4**). *Rickettsia africae* was detected in all tick tissues collected from *Am. gemma* and *Rh. pulchellus*. *Rickettsia africae* DNA detection rate was highest in the MG (41.7%) of *Hy. dromedarii*, absent from all HL samples and present in the SL (5.6%) and SGs (2.8%) of only two male ticks (**Figure 6A**). In contrast, detection rates of *R. africae* in *Am. gemma* were highest in the SL (42.5%) and HL (45%) (**Figure 6A**) (**Supplementary Table 1**). Though *R. africae* was not detected in *Hy. rufipes*, *R. aeschlimannii* was detected in all tissues of *Hy. rufipes* ticks (**Table 4**), but in none of the tissues of the other tick species (**Figure 6B**). *Ehrlichia ruminantium* was detected only in Marsabit County at high detection rates in all species collected – *Rh. pulchellus*, *Am. gemma*, *Hy. rufipes*, and *Hy*. *dromedarii* (**Table 4**) (**Figure 6C**). However, the *E. ruminantium* detection rates were highest in all tissues of *Am. gemma* (SL: 15%, HL: 20%, SGs: 20%, MG: 22.5%). We detected the co-infections of *R. africae* and *E. ruminantium* in tick tissues of *Am. gemma*, *Rh. pulchellus*, and *Hy. dromedarii*, and co-infections of *R. aeschlimannii* and *E. ruminantium* in *Hy. rufipes* (**Supplementary Table 2**).

**Figure 7:**
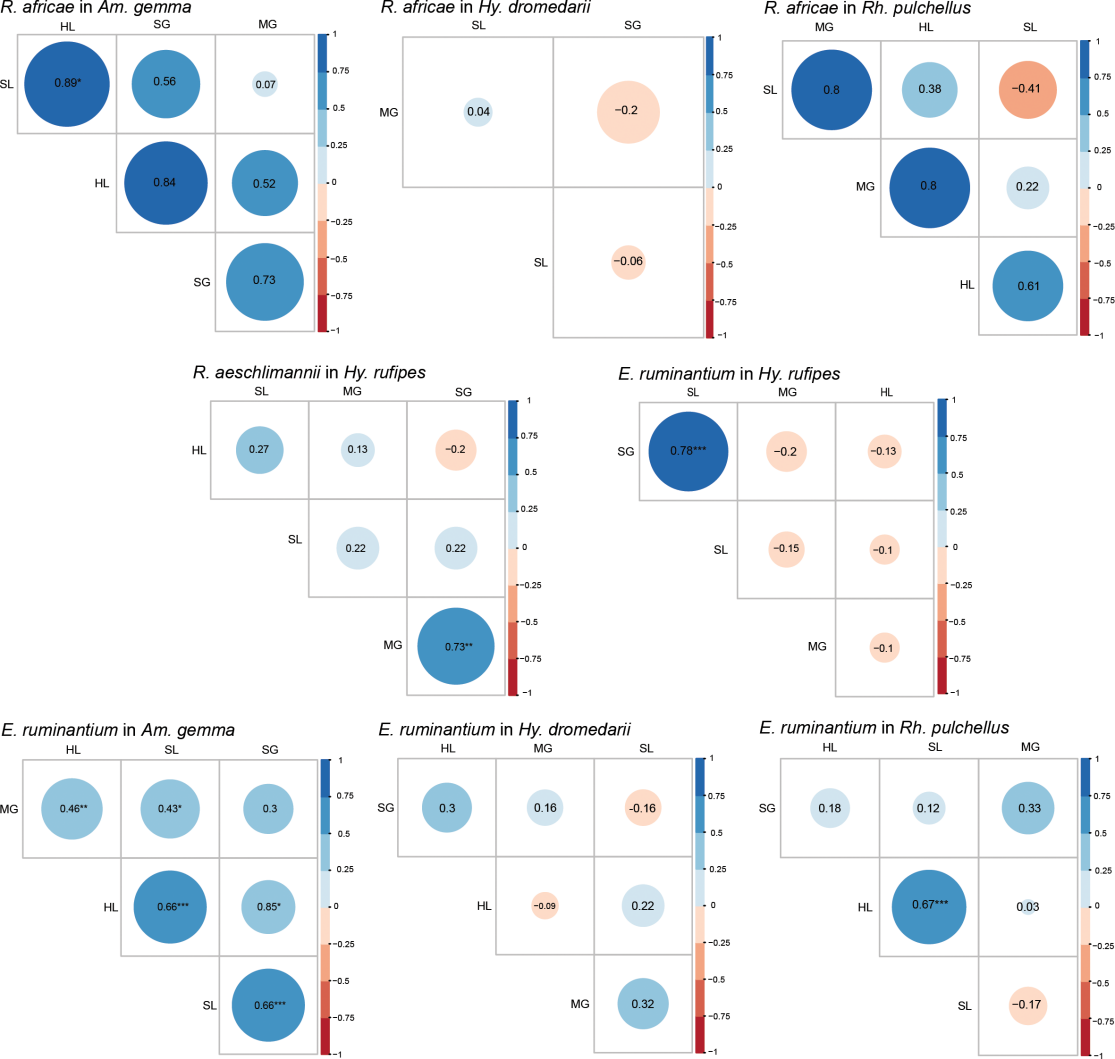
Pearson correlation matrix heat map summarizing correlations of pathogen, *R. africae*, *R. aeschlimannii*, and *E. ruminantium*, occurrence and tissue type of infected individuals for different tick species. The different colors represent Pearson correlation coefficients, and the different circles sizes represent the P-value (*P < 0.05, **P < 0.01, ***P < 0.001). SL, saliva: HL, hemolymph; SG, salivary glands; MG, midgut.

We performed Pearson correlation analysis for *R. africae* and *E. ruminantium* infections in various tick tissues across the four tick species. In *Am. gemma*, we noted that *R. africae* in HL was significantly correlated with its occurrence in SL (P < 0.05). While in *E. ruminantium* infected *Am gemma*, we recorded significant correlations between the HL and SL (P < 0.001), SL and SGs (P < 0.001), MG and HL (P < 0.01), SL and MG (P < 0.05), and the HL and SGs (P < 0.05) (**Figure 7**). *Ehrlichia ruminantium* occurrence was similarly correlated between the HL and SL (P < 0.05) in *Rh. pulchellus* and the SL and SGs in *Hy. rufipes* (P < 0.001) (**Figure 7**). In *Hy. rufipes*, we observed that *R. aeschlimannii* in the SGs was significantly correlated to its occurrence in MG (P < 0.01).

## 4 Discussion

We detected for the first time TBPs within various tick tissues (saliva, hemolymph, salivary glands, and midgut) of *Am. gemma*, *Rh. pulchellus*, *Hy. dromedarii*, and *Hy. rufipes* collected from camels, when attempting to identify potential mechanisms of pathogen transmission and to disentangle infection status from likely vector competence. *Rhipicephalus pulchellus* was the most abundant tick species in Badassa and Mpala, where camel herds were closely associated with cattle. *Rhipicephalus pulchellus* has been identified previously in high numbers in camels closely associated with cattle, which are considered to be the main hosts of this tick species (Dioli, 2002). However, in Laisamis, we only collected *Hyalomma* species, with *Hy. rufipes* being more abundant than *Hy. dromedarii*. Unexpectedly, in Mpala Research Centre, only one female of *Hy. rufipes* was collected, while no *Hy. dromedarii* were found. The absence of *Rh. pulchellus* and *Am. gemma* in Laismais might be due to less vegetation cover, lower altitude, lower relative humidity, and higher temperature compared to Badassa. Conversely, the higher prevalence of *Hy. dromedarii* and *Hy. rufipes* in Laisamis could be attributed to the fact that *Hyalomma* spp. can survive in harsh environments (Hoogstraal et al., 1964; Perveen et al., 2020). Temperature and relative humidity are known to impact host-seeking behavior and survival rates (Parola and Raoult, 2001; Ruiz-Fons et al., 2012). Similar to the findings of Getange et al. (2021) and Mwamuye et al. (2017), we were not able to obtain CO1 and 16S rRNA sequences from some tick species. These markers may not be suitable for the molecular identification of ticks, in contrast to those obtained from 12S rRNA, which were all successfully amplified.

The higher infection rate of *Ca*. A. camelii recorded in camels in Laisamis could be attributed to the higher infestation rates with camel keds, *Hippobosca camelina*, a species which has been identified as a potential vector of *Ca.* A. camelii (Bargul et al., 2021). The camels were apparently healthy, which is in line with previous studies (Bastos et al., 2015; Kidambasi et al., 2020; Getange et al., 2021; Younan et al., 2021). This carrier status of the camels plays a crucial role in maintaining successful bacteria transmission (Brown, 2012).

*Anaplasma phagocytophilum*, some strains of which can cause pathogenic anaplasmosis in humans (Chen et al., 1994) or domestic ruminants (Gordon et al., 1932), and other *Anaplasma* spp. (i.e., *A. marginale, A. platys*, and *A. ovis*) were detected in apparently healthy camels. *Anaplasma* spp., including *A. phagocytophilum*, have previously been detected in camels from Saudi Arabi (Alanazi et al., 2020), Algeria (Bessas et al., 2022), and the United Arab Emirates (El Tigani-Asil et al., 2021). Further, in Ben Said et al. (2013) found 29% of tested camels to be seropositive for *A. phagocytophilum*. Though it remains unknown if camels are definitive or reservoir hosts of *A. phagocytophilum* (Selmi, 2022) or whether the strains found in camels are also pathogenic, our findings strongly support the epidemiological role of camels in their spread and emergence.

Moreover, we detected for the first time *E. chaffeensis*, the etiological agent of human monocytic ehrlichiosis (Paddock and Childs, 2003; Rikihisa, 2022), as well as *Ca.* E. regneryi, in two camels in Badassa region. Recent research in North Kenya, conducted by Getange et al. (2021), identified *E. chaffeensis* in *Amblyomma lepidum* ticks collected from camels. Previous studies have reported the presence of this pathogen in various animal species, including dogs, coyotes, goats, and white-tailed deer (Kocan et al., 2000; Davidson et al., 2001; Dugan et al., 2005; Liu et al., 2022). In Kenya, *Ca*. E. regneryi has been identified in both the blood of camels and associated *Hyalomma* ticks (Getange et al., 2021). However, we did not detect the pathogen in any of the tick species examined. Further investigations of the asymptomatic carrier status of *Ca.* A. camelii, *Ca.* E. regneryi and *E. chaffeensis* are needed to better understand their epidemiology, vector involvement, and zoonotic potential, which can contribute to improved control, prevention strategies and mitigate the risk associated with these emerging bacteria.

The high detection rates of *R. africae*, the causative agent of African tick bite fever (ATBF) (Mazhetese et al., 2019), were observed across all tissues of *Am. gemma*, mostly in the HL and least in the MG. In contrast, *R. africae* was detected at a lower rate in *Rh. pulchellus*, in which the highest detection rate was in the MG and lowest in the HL. Interestingly, *R. africae* was detected at highest rates in the MG of *Hy. dromedarii*, in which it was entirely absent in the HL. It is known that *Rickettsia* spp. invade the SGs from the MG via the HL (Socolovschi et al., 2009; Liu and Bonnet, 2014). The complete absence of *R. africae* in the HL of *Hy. dromedarii* suggests that the pathogen was unable to cross the MG barrier to SL presumably due to physical barrier or innate immunity.

Though *R. africae* has been associated with *Hy. dromedarii* ticks based on whole-tick analyses (Kernif et al., 2012), our findings suggest that such associations may be due to the pathogen’s presence in the midgut from bloodmeals, from which it may not succeed in crossing the MG barrier into the HL, making *Hy. dromedarii*’s involvement in *R. africae* transmission, unlikely. Similarly, though *R. africae* has also previously been associated with *Rh. pulchellus* ticks (Mutai et al., 2013), the low levels of *R. africae* in its HL compared to its MG found in this study suggests that *Rh. pulchellus* may be at most an inefficient vector of *R africae*. A similar phenomenon was observed by Bremer et al. (2005) for *Ehrlichia canis*, where acquisition and transmission occurred in *Rh. sanguineus* males in the absence of females. Similarly, we detected *R. aeschlimannii* at a high rate in the HL and at a low rate in the MG of *Hy. rufipes*. Based on these results, we proposed that HL could be a crucial indicator for assessing vector competency. However, previous studies in Kenya, have reported *R. aeschlimannii* in *Hy. dromedarii*, *Hy. impeltatum*, *Hy. truncatum*, *Am. gemma*, and *Rh. pulchellus* (Koka et al., 2017; Getange et al., 2021). Further studies are recommended to explore the factors that contribute to the vectorial capacity in the transmission of *R. aeschlimannii* exclusively by *Hy. rufipes* and to determine the limitation in the other ticks. In this, study, we could not rely on the SGs and SL as indicators of vector competence, as we detected some TBPs in saliva, but not in the salivary glands, and vice versa. This could be due to uncertainty about whether the pathogen detected in SGs originated from the previous nymphal stage or those in the SL resulted from regurgitate of the MG.

*Candidatus* A. camelii was not detected in any tick species collected from camels, which is not in line with a study conducted by Getange et al. (2021) in North Kenya, who found the pathogen in various tick species such *Am. lepidum*, *Hy. rufipes*, *Hy. dromedarii* and *Rh. pulchellus* (Getange et al., 2021). As mentioned above, the *Ca.* A. camelii can be transmitted by camel keds (Bargul et al., 2021), which were highly abundant, particularly in Laisamis, during our sampling. It is worth stating that the method of tick collection in the other studies, which requires preserving ticks in liquid nitrogen or alcohol immediately after detaching the tick from the host might not provide a comprehensive understanding of pathogen transmission by the vector. We transported collected ticks to the laboratory, keeping them alive for more than one week, which may have allowed the ticks to clear the pathogen. More studies are needed to understand the tripartite interactions between hosts, vectors, and pathogens, especially the factors that influence the selective acquisition of pathogens by specific vectors.

We identified *Ehrlichia ruminantium*, the causative agent of heartwater disease (Postigo et al., 2007), at higher rates in all *Am. gemma* tissues than in *Hy. rufipes*, *Rh. pulchellus* and *Hy. dromedarii*. *Ehrlichia ruminantium* is known to be transmitted by *Amblyomma* ticks (Postigo et al., 2007). The high detection rates and positive correlation among all *Am. gemma* tissues provide further evidence suggesting that *Am. gemma* is a principal vector of *E. ruminantium* in North Kenya. This is consistent with previous findings implicating *Amblyomma* species as major vectors of *E. ruminantium* (Wesonga et al., 2001; Kelly et al., 2011; Tomassone et al., 2012; Esemu et al., 2013; Getange et al., 2021; Younan et al., 2021). Furthermore, we found higher detection rates of *E. ruminantium* in the HL of *Rh. pulchellus* compared to the HL of both *Hy. dromedarii* and *Hy. rufipes*, suggesting that the pathogen may disseminate and replicate with greater success within *Rh. pulchellus* tissues. Previous studies confirm the circulation of *E. ruminantium* in *Rh. pulchellus* ticks (Tomassone et al., 2012; Omondi et al., 2017; Mucheka et al., 2023). Indeed, further studies are needed to assess the vectorial capacity of *Rh. pulchellus* in transmitting *E. ruminantium*. For successful transmission, the pathogen must interact, replicate, and disseminate through tick organs. The absence of the *Rickettsia* spp. and *E. ruminantium* infection in the blood of camels might be attributed to the pathogens’ ability to replicate within the tick bite site as well as within the endothelial cells of blood vessels and the host organs (Prozesky, 1987; Kim, 2022).

## 5 Conclusions

Screening of TBPs in the finer organ scale assists in identifying the mechanisms of pathogen transmission and in the disentanglement of infection status from likely vector competence. Although TBPs can be found in both animals and attached ticks, the presence of TBPs in tick bodies does not necessarily indicate that ticks are efficient vectors for pathogen transmission. Our findings suggest that detection of the pathogen in the hemolymph could serve as an indicator of vector competence. Understanding the distribution of TBPs within tick tissues widens our knowledge of TBD epidemiology, particularly the potential of specific tick species to act as competent vectors or dead-end hosts. Further studies are needed to investigate the factors affecting the tick’s vectorial capacity. We recommend investigating the asymptomatic carrier status of camels infected with *Ca.* A. camelii, *Ca.* E. regneryi, and *E. chaffeensis* to enhance understanding of their epidemiology, vector involvement, and zoonotic potential, which could improve control strategies and reduce associated risks. Knowledge on the prevalence and the geographical distributions of tick species and their potential vector competence is important for the effective management and control of TBDs.

## Data availability statement

The datasets presented in this study can be found in online repositories. The names of the repository/repositories and accession number(s) can be found below: https://www.ncbi.nlm.nih.gov/genbank/, OR138025-OR138032, https://www.ncbi.nlm.nih.gov/genbank/, OR136390-OR136395, https://www.ncbi.nlm.nih.gov/genbank/, OR123453-OR123456, https://www.ncbi.nlm.nih.gov/genbank/, OR136355-OR136357, https://www.ncbi.nlm.nih.gov/genbank/, OR136371-OR136378, https://www.ncbi.nlm.nih.gov/genbank/, OR130207- OR130215.

## Ethics statement

The animal studies were approved by Pwani University Ethics Review Committee. The studies were conducted in accordance with the local legislation and institutional requirements. Written informed consent was not obtained from the owners for the participation of their animals in this study because prior to livestock sampling, verbal and non-written consent was obtained from the livestock keepers, as most were unable to read or write. Field assistants from the community assisted in restraining the camels and helped in translating the language from English to the local language spoken by the community members to ensure that they understood the purpose of the study and how it would benefit them.

## Author contributions

RK: Conceptualization, Data curation, Formal analysis, Funding acquisition, Investigation, Methodology, Project administration, Resources, Software, Supervision, Validation, Visualization, Writing – original draft, Writing – review & editing. AB: Conceptualization, Supervision, Writing – review & editing. JB: Investigation, Writing – review & editing. DG: Data curation, Investigation, Methodology, Writing – review & editing. JK: Investigation, Methodology, Writing – review & editing. DM: Conceptualization, Supervision, Writing – review & editing. JV: Conceptualization, Data curation, Formal analysis, Funding acquisition, Investigation, Methodology, Project administration, Resources, Software, Supervision, Validation, Visualization, Writing – original draft, Writing – review & editing.
