# Supplementary material for: Tissue-specific localization of tick-borne pathogens in ticks collected from camels in Kenya: insights into vector competence
Source: Front Cell Infect Microbiol. 2024 Apr 18;14:1382228. doi: 10.3389/fcimb.2024.1382228 (PMC11063324; doi:10.3389/fcimb.2024.1382228)
Supplement: Supplementary file 1 [file Table_1.docx]

Supplementary Material

**Supplemetary Table 1:** **Detected tick-borne pathogens in tick tissues collected from camels.**

| Bacterial pathogen | Tick species | Number of infected ticks and tissues (Detection rate, %) | | | |
| --- | --- | --- | --- | --- | --- |
|  |  | **SL** | **HL** | **SGs** | **MG** |
| *Rickettsia africae* | *Am. gemma* | **17**  (42.5%) | **18**  (45%) | **11**  (27.5%) | **11**  (27.5%) |
|  | *Rh. pulchellus* | **3**  (8.8%) | **4**  (11.8%) | **3**  (8.8%) | **6**  (17.6%) |
|  | *Hy. dromedarii* | **2**  (5.6%) | **0** | **1**  (2.8%) | **15**  (41.7%) |
| *Rickettsia asechlimannii* | *Hy. rufipes* | **9**  (56.3%) | **12**  (75%) | **7**  (43.8%) | **7**  (43.8%) |
| *Ehrlichia ruminantium* | *Am. gemma* | **6**  (15 %) | **8**  (20%) | **8**  (20%) | **9**  (22.5%) |
|  | *Rh. pulchellus* | **2**  (5.9%) | **4**  (11.8%) | **7**  (20.6%) | **5**  (14.7%) |
|  | *Hy. dromedarii* | **6**  (16.7%) | **2**  (5.6%) | **4**  (11.1%) | **4**  (11.1%) |
|  | *Hy. rufipes* | **2**  (12.5%) | **1**  (6.3%) | **3**  (18.8%) | **2**  (12.5%) |

SL: saliva; HL: haemolymph, SG: salivary glands; MG: midgut

**Supplementary Table 2: Number of co-infections of tick-borne pathogens in tick tissues collected from camels**.

| **Tick**  **species** | **Number of co-infections detected in ticks (co-infection rate, %)** | | |  | **Number of co-infections detected in tick tissues. (co-infection rate, %)** | | | | | **Co-infected bacteria** |
| --- | --- | --- | --- | --- | --- | --- | --- | --- | --- | --- |
|  | |  | | **SL** | | **HL** | **SG** | **MG** |  | |
| ***Am. gemma*** | | | **8** (20%) | **6** (15%) | | **7** (17.5%) | **4** (10%) | **2** (5%) | *R. africae* & *E. ruminantium* | |
| ***Rh. pulchellus*** | | | **9** (26.5%) | **2** (5.9%) | | **3** (8.8%) | **2** (5.9%) | **5** (14.7%) | *R. africae* & *E. ruminantium* | |
| ***Hy. dromedarii*** | | | **5** (13.9%) | **1** (2.8%) | | **0** | **1** (2.8%) | **3**(8.3%) | *R. africae* & *E. ruminantium* | |
| ***Hy. rufipes*** | | | **6** (16.7%) | **3** (18.8%) | | **1** (6.3%) | **2** (12.5%) | **3** (18.8%) | *R. aeschlimannii* & *E. ruminantium* | |

SL: saliva; HL: haemolymph, SG: salivary glands; MG: midgut
